# Integrating peripheral blood and brain transcriptomics to identify immunological features associated with Alzheimer’s disease in mild cognitive impairment patients

**DOI:** 10.3389/fimmu.2022.986346

**Published:** 2022-09-09

**Authors:** Xiao-hang Qian, Xiao-li Liu, Sheng-di Chen, Hui-dong Tang

**Affiliations:** ^1^ Department of Neurology and Institute of Neurology, Rui Jin Hospital, Shanghai Jiao Tong University School of Medicine, Shanghai, China; ^2^ Department of Neurology, Shanghai Fengxian District Central Hospital, Shanghai Jiao Tong University Affiliated Sixth People’s Hospital South Campus, Shanghai, China; ^3^ Medical Center on Aging of Ruijin Hospital, Shanghai Jiao Tong University School of Medicine, Shanghai, China

**Keywords:** mild cognitive impairment, Alzheimer’s disease, peripheral immune cells, immune hub gene, RELA, HSP90AA1

## Abstract

**Background:**

Immune system dysfunction has been proven to be an important pathological event in Alzheimer’s disease (AD). Mild cognitive impairment (MCI), as a transitional stage between normal cognitive function and AD, was an important research object for the screening of early diagnostic markers and therapeutic targets for AD. However, systematic assessment of peripheral immune system changes in MCI patients and consistent analysis with that in the CNS were still lacking.

**Methods:**

Peripheral blood transcriptome data from the AddNeuroMed Cohort (*n* = 711) was used as a training dataset to assess the abundance of 24 immune cells through ImmuCellAI and to identify MCI-related immune signaling pathways and hub genes. The expression level of the immune hub gene was validated in peripheral blood (*n* = 587) and brain tissue (78 entorhinal cortex, 140 hippocampi, 91 temporal cortex, and 232 frontal cortex) validation datasets. Finally, reliable immune hub genes were applied for Gene Set Enrichment Analysis and correlation analysis of AD pathological characteristics.

**Results:**

MCI patients have early changes in the abundance of various types of immune cells in peripheral blood, accompanied by significant changes in NF-kB, TNF, JAK-STAT, and MAPK signaling pathways. Five hub immune-related differentially expressed genes (NFKBIA, CD4, RELA, CASP3, and HSP90AA1) were screened by the cytoHubba plugin in Cytoscape and the least absolute shrinkage and selection operator (LASSO) regression. Their expression levels were significantly correlated with infiltration score and the abundance of monocytes, natural killer cells, Th2 T cells, T follicular helper cells, and cytotoxic T cells. After validation with independent datasets derived from peripheral blood and brain, RELA and HSP90AA1 were identified as two reliable immune hub genes in MCI patients and had consistent changes in AD. The Gene Set Enrichment Analysis (GSEA) showed that their expression levels were closely associated with Alzheimer’s disease, JAK-STAT, calcium signaling pathway, etc. In addition, the expression level of RELA was positively correlated with β- and γ-secretase activity and Braak stage. The expression level of HSP90AA1 was negatively correlated with α- and β-secretase activity.

**Conclusion:**

Immune system dysfunction was an early event in AD. It provides a new target for the early diagnosis and treatment of AD.

## Introduction

Alzheimer’s disease (AD) is a progressive age-related neurodegenerative disease and is the leading cause of dementia (1). Because of its unclear pathogenesis and lack of disease-modifying therapy, it places a huge burden on patients, their families, and the healthcare system around the world. Its classic pathophysiological hallmarks include severe neuronal loss together with senile plaques of extracellular amyloid-beta (Aβ) aggregation and neurofibrillary tangles (NFT) of intracellular Tau hyperphosphorylation (2). Over the past decade, AD therapeutic research targeting the core pathologies has been accelerated, but satisfactory results have not been achieved (3). This suggests that the AD research strategy should be re-examined. Recently, dysfunction of the immune system has been identified as an important event in the progress of AD (4). A series of convincing experimental and clinical studies have confirmed that the immune system changes dynamically at different stages of AD and plays a multifaceted role (5). The immune system can be divided into the central immune system and peripheral immune system. They have close interaction with each other. In the central nervous system (CNS), microglia are the resident immune cells, and abnormal activation of microglia has been identified as an early event of AD (6). In addition, immune cells of peripheral origin, including adaptive and innate immune cells, can also participate in the AD process in multiple ways, such as being recruited into the CNS to participate in immune regulation and producing Aβ-targeted antibodies (4). Therefore, early diagnosis and treatment strategies targeting the immune system have great prospects for clinical application in the future.

Mild cognitive impairment (MCI) due to AD is considered to be a transitional phase between normal cognitive function and AD (7). It is defined as the stage that an objective cognitive impairment has no effect on activities in daily life (8). However, patients at this stage can already detect pathological changes similar to AD, such as accumulation of Aβ and p-Tau (9). Therefore, this stage will provide an important reference value for the development of AD-related diagnostic markers and therapeutic targets. Similarly, previous studies have found changes in the immune system in MCI patients. For example, patients with MCI were found to have moderate neuroinflammation, compared with healthy controls (HC) through positron emission tomography (PET) imaging targeting 18 kDa translocator protein (TSPO) ligands (10). In recent years, some studies have reported the association between peripheral immune cells and AD, but it was limited to a few indicators, including platelet-to-lymphocyte ratio (PLR), granulocyte-to-lymphocyte ratio (GLR), lymphocyte-to-monocyte ratio (LMR), and systemic immune-inflammation index (SII) (11, 12). Systematic assessment of the abundance differences of various peripheral immune cells among HC, MCI, and AD patients and their consistency with the trend of the immune system in the CNS is still lacking.

This study was based on peripheral blood transcriptome expression data from large clinical samples of HC, MCI, and AD patients (*n* = 711). ImmuncellAI bioinformatics software was used to perform a detailed assessment of 24 types of peripheral immune cell abundance. On this basis, we further analyzed the changes in immune signaling pathways and immune hub genes in MCI patients and verified their expression levels in peripheral blood and the brain through independent clinical cohort samples. Finally, the potential mechanism of immune hub genes and their correlation with the pathological features of AD were explored. This study will provide an important target for the early diagnosis and treatment of AD at the MCI stage.

## Materials and methods

### Data collection and processing

Gene expression profiles from peripheral whole blood were downloaded from the NCBI Gene Expression Omnibus public database (GEO, https://www.ncbi.nlm.nih.gov/geo/). The GSE63060 and GSE63061 data files originated from the EU-funded AddNeuroMed Cohort, a large cross-European AD biomarker study based on peripheral blood RNA (13). A total of 238 HC, 189 MCI, and 284 AD patients were included in GSE63060 and GSE63061 as the training datasets for this study. In addition, the GSE140829 data file, including 249 HC, 134 MCI, and 204 AD patients’ peripheral blood gene expression data, was used as the validation dataset. The AlzData database is an integrated high-throughput database, including transcriptomes, proteomics, and genomics (GWAS and Whole Exome Sequencing) (14). Transcriptome data from four brain regions (entorhinal cortex, hippocampus, temporal cortex, and frontal cortex) in the Alzadata database were used as validation datasets to verify the expression level of the immune hub gene in the brain. The GSE106241 was a gene expression profile containing 60 temporal cortical tissue samples with different AD-related neurofibrillary pathology. Combining α-, β-, and γ-secretase activity with Aβ42 levels, the GSE106241 was applied to determine the correlation between the expression level of immune hub genes and AD-related pathology. The flow chart of this study is shown in (**Figure S1**).

### Immune cell infiltration abundance analysis

The Immune Cell Abundance Identifier (ImmuCellAI, http://bioinfo.life.hust.edu.cn/web/ImmuCellAI/) was an online tool for accurately estimating the abundance of 24 immune cells based on the RNA sequencings and microarray datasets from blood or tissue samples (15). A total of 24 immune cells contain 18 T-cell subsets (CD4^+^, CD4^+^ naïve, induced regulatory T (iTreg), natural regulatory T (nTreg), type 1 regulatory T (Tr1), Th1, Th2, Th17, T follicular helper (Tfh), central memory T (Tcm), effector memory T (Tem), CD8^+^, CD8^+^ naïve, cytotoxic T (Tc), mucosal-associated invariant T (MAIT), exhausted T (Tex), gamma delta T (γδ T), and natural killer T (NKT) cells] and six other important immune cells (monocytes, macrophages, neutrophils, B cells, DC, and NK cells). The ImmuCellAI was used to estimate the difference in peripheral immune cells between the HC, MCI, and AD groups. The immune infiltration score represents a total percentage abundance of 24 immune cells.

### Construction of weighted correlation network analysis

Based on the genetic and clinical data in GSE63060 and GSE63061, the Weighted Correlation Network Analysis (WGCNA) package in R was used to construct a weighted mRNA coexpression network. At first, the Median Absolute Deviation (MAD) of each gene was calculated based on the gene expression spectrum, and 50% of the minimum MAD genes were removed. The scale-free coexpression network was then built. Under an appropriate power (*β* = 8), the adjacency matrix was transformed into the topological overlap matrix (TOM). Thirdly, hierarchical clustering was performed to identify modules, and the eigengene was calculated. Finally, the correlation between the MCI group and each module was calculated through Pearson’s correlation analysis.

### Identification of differentially expressed genes

The differentially expressed genes (DEGs) between HC and MCI group were identified through the “limma” package in R. The statistical significance value of the obtained differential genes was set as *p*-value < 0.05 and |log2 Fold change| > 0. The volcano plot was produced by the “ggplot2” package in R to visualize the total DEGs between HC and MCI. The top15 DEGs with the most significant upregulation and downregulation was then shown in a volcanic map by using “Pheatmap” package in R. The Venn diagram was used to screen and visualize the common immune-related DEGs of WGCNA, DEGs, and immunity genes from InnateDB (https://www.innatedb.com/) and ImmPort (https://www.immport.org/home) databases (16, 17).

### Gene ontology functional, Kyoto encyclopedia of genes and genomes pathway enrichment, and protein-protein interaction network analyses

To further clarify the potential functional annotation and pathway enrichment associated with the DEGs and hub genes, Gene Ontology (GO) analyses, including cellular component (CC), biological process (BP), molecular function (MF), and Kyoto Encyclopedia of Genes and Genomes (KEGG) pathways, were performed to figure out the functional roles of Robust DEGs by the “clusterProfiler package” in R (significant as a *p*-value of < 0.05 and an FDR of < 0.25). The protein–protein (PPI) network was constructed through the NetworkAnalyst with a 900 confidence score. (https://www.networkanalyst.ca/) (18).

### Immune-related hub DEG selection

The cytoHubba plugin in Cytoscape was applied to screen out immune-related hub genes through three algorithms, including Edge Percolated Component (EPC), Maximum Neighborhood Component (MNC), and Maximal Clique Centrality (MCC). The top 15 immune-related DEGs obtained by the three algorithms were selected, and the common part was selected for the least absolute shrinkage and selection operator (LASSO) regression. The expression of nine hub genes was analyzed using LASSO regression with a binomial model and a lambda value equal to the minimum mean cross-validated error by using the “glmnet” package in R.

### Gene set enrichment analysis

In this part, we used Gene Set Enrichment Analysis (GSEA) to analyze the potential molecular mechanism of core gene immunity in AD. The expression profile data of 189 MCI in the training dataset were selected and divided into high and low groups according to the expression level of immune hub DEGs. The annotated gene set c2.cp.kegg.v7.1.symbols.gmt was selected as a reference dataset reference gene list. The *p*-value < 0.05 was set as the cutoff value for the GSEA.

### Statistical analysis

GraphPad Prism 5.0 software and Sangerbox online software (http://sangerbox.com/) were used for statistical analysis and graphing. Bonferroni correction and Duncan’s multiple range test were used for multiple testing correction. The distribution of continuous variables between three groups was analyzed through a one-way analysis of variance (ANOVA), followed by Fisher’s least significant difference (LSD) test or Kruskal–Wallis test, followed by Dunn’s multiple comparisons test. The difference analysis between the two groups was completed by a two-tailed Student’s *t*-test or Mann–Whitney test. The value of statistical difference was set as *p* < 0.05 (^*^ *p* < 0.05; ^**^ *p* < 0.01; ^***^ *p* < 0.001; ns, no statistical difference.

## Results

### MCI patients showed similar peripheral immune cell alteration to AD

**Figure 1:**
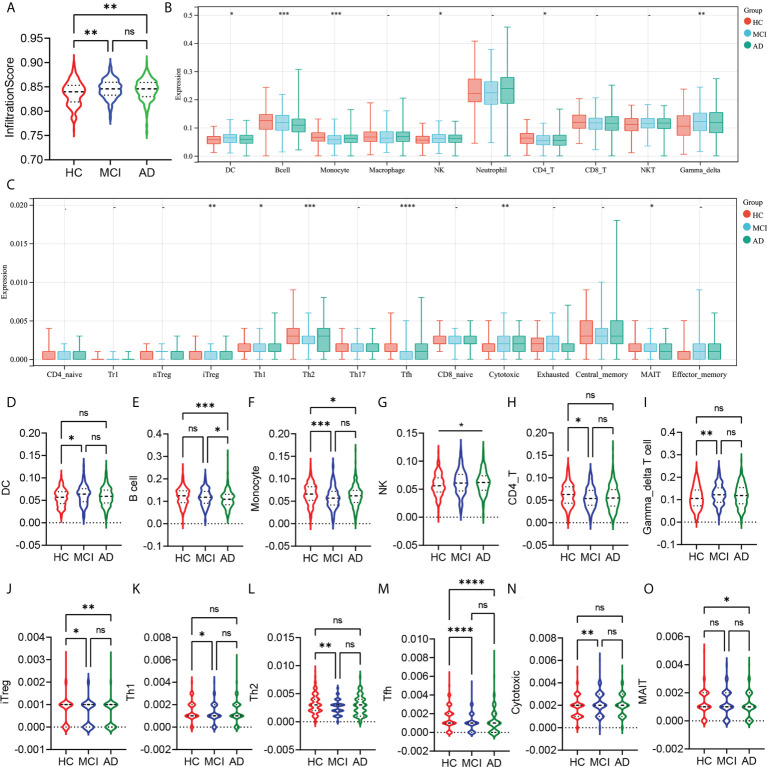
Analysis of the peripheral immune cells’ abundance differences between HC, MCI, and AD groups. **(A)** The **i**nfiltration score difference between HC, MCI, and AD groups. **(B)** The abundance difference of DC, B cells, monocytes, macrophages, NK, neutrophils, CD4 T cells, CD8 T cells, NKT, and γ-δ T cells between the HC, MCI, and AD groups. **(C)** The abundance difference of CD4^+^ naïve, induced regulatory T (iTreg), natural regulatory T (nTreg), type 1 regulatory T (Tr1), Th1, Th2, Th17, T follicular helper (Tfh), central memory T (Tcm), effector memory T (Tem), CD8^+^ naïve, cytotoxic T (Tc), mucosal-associated invariant T (MAIT), and exhausted T (Tex) between HC, MCI, and AD groups. **(D–O)** Violin plots show statistically different immune cell abundances between HC, MCI, and AD. The red color represents the HC group, the blue color represents the MCI group, and the green color represents the AD group. *p*-values between each group are shown at the top of the figure. HC, healthy control; MCI, mild cognitive impairment; AD, Alzheimer’s disease. *p* < 0.05 was considered to be statistically significant. ^*^ *p* < 0.05; ^**^ *p* < 0.01; ^***^ *p* < 0.001; ****p<0.0001; ns, no statistical difference.

First, we analyzed the differences in the abundance of 24 types of immune cells in the peripheral blood of HC, MCI, and AD patients. The infiltration score represented a combination of the percentage abundance of 24 immune cells. The results showed that the infiltration score of the MCI and AD groups were significantly higher than the HC group, but there was no difference between the MCI and AD groups (**Figure 1A**). Among the 10 immune cell subtypes, the abundance of DC, NK, and γ-delta T cells was increased, and the abundance of monocyte and CD4 T cell was reduced in the MCI group compared with the HC group (**Figures 1B, D, F–I**). Similar trends were observed in AD patients, although there were no statistically significant differences. In addition, we found that the B cell was obviously decreased in the AD group, compared with the HC and MCI group (**Figure 1E**). The abundance of macrophages, neutrophils, CD8 T cells, and NKT showed no statistically significant difference between the three groups (**Figure 1B**). In the subtypes of CD4 and CD8 T cells, there was a lower abundance of iTreg and Tfh in the MCI and AD groups, compared with the HC group (**Figures 1C, J, M**). The abundance of Th1 and Th2 was only decreased in the MCI group, compared with the HC group (**Figures 1K, L**). At the same time, the MCI group has a higher abundance of cytotoxic T cells than the HC group (**Figure 1N**). In addition, the abundance of MAIT was decreased in the AD group, compared with the HC group (**Figure 1O**).

### Integration of WGCNA and differential gene analysis screens out MCI-associated immune genes

**Figure 2:**
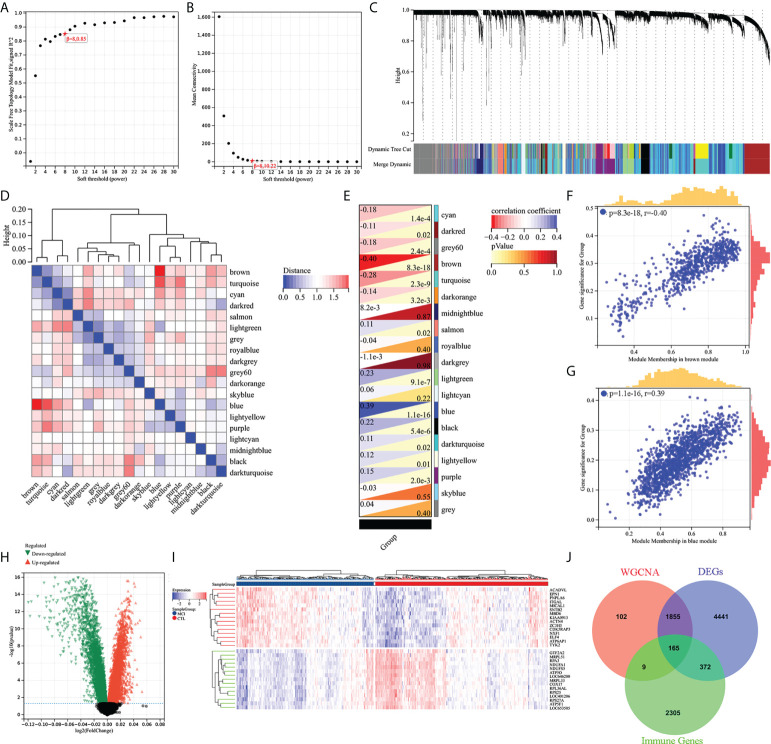
Screening of immune-related different expression genes (IRDEGs). **(A)** Analysis of the scale-free index for various soft-threshold powers (β). **(B)** Analysis of the mean connectivity for various soft-threshold powers. **(C)** Recognition module, each module was given an individual color as an identifier, including 19 different modules. **(D)** Coexpression similarity of entire modules based on hierarchical clustering of module eigengenes and correlation between different modules; red indicates high adjacency (positive correlation), and blue indicates low adjacency (negative correlation). **(E–G)** Correlation heat map of gene modules and phenotypes, with blue being positively correlated with the phenotype and red being negatively correlated. **(H)** Volcanic map of differential expression genes between the HC and MCI groups. *p*-value < 0.05 and |log2 Fold change| > 0. **(I)** The heat map shows the top 15 differentially expressed genes that are most significantly up- or downregulated. **(J)** The Venn diagram represents the common intersection of WGCNA, differentially expressed genes, and immunity genes from the InnateDB and ImmPort database.

WGCNA and differential gene analysis were applied to screen out MCI-associated immune genes. Firstly, we selected the top 50% highest variance of the expression profile (a total of 8,176 genes) from 427 samples (238 HC and 189 MCI) for WGCNA analysis. We then construct the scale-free network with a *β*-value equal to 8 (*R* ^2^ = 0.85) (**Figures 2A, B**). Finally, a total of 19 coexpression modules were constructed (**Figure 2C**). A 19 coexpression module feature vector cluster was presented (**Figure 2D**). To further analyze the association between the models and phenotype, we calculated the correlation coefficients of each model with the MCI group. We found that the brown module (*r* = −0.40, *p* = 8.3e−18) was the most negatively related to the MCI group, while the blue module (*r* = 0.39, *p* = 1.1e−16) was the most positively related (**Figures 2E–G**). The 2,131 genes from brown and blue modules were selected for the next analysis. In addition, the DEGs between the HC and MCI groups were analyzed. Under the screening conditions of *p*-value < 0.05 and |log2 Fold change| > 0, a total of 3,608 upregulated and 3,225 downregulated genes were identified (**Figure 2H**). The top 15 differentially upregulated and downregulated genes were displayed on the heat map (**Figure 2I**). Finally, we integrated WGCNA, DEGs, and immunity genes from the InnateDB and ImmPort databases to screen 165 immune-related differentially expressed genes (IRDEGs) that were significantly associated with the MCI group (**Figure 2J**; **Supplementary Table S1**).

### Enrichment analysis of IRDEGs and protein–protein interaction network analysis

**Figure 3:**
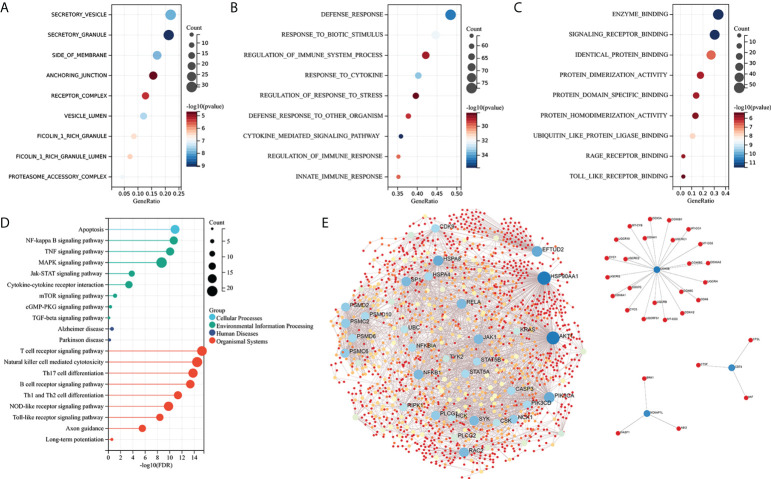
Enrichment analysis and PPI network construction of IRDEGs. **(A–C)** GO function enrichment analysis (cellular components, biological process, and molecular function). **(D)** KEGG pathway enrichment analysis. **(E)** PPI network of IRDEGs. GO, Gene Ontology; KEGG, Kyoto Encyclopedia of Genes and Genomes; PPI, protein–protein interaction; IRDEGs, immune-related different expression genes.

GO functional and KEGG enrichment analyses were performed on IRDEGs to elucidate their molecular function. The cellular component analysis revealed that the IRDEGs are mainly secretory vesicles, secretory granules, the side of the membrane, anchoring junction, etc. (**Figure 3A**). Their biological processes were involved in defense responses, responses to biotic stimulus, regulation of immune system processes, responses to cytokines, etc. (**Figure 3B**). The molecular function of the analysis showed that IRDEGs were related to enzyme binding, signaling receptor binding, identical protein binding, etc. (**Figure 3C**). The KEGG enrichment analysis of IRDEGs showed that they were involved in the MAPK signaling pathway, NF-kB signaling pathway, Alzheimer’s disease, T-cell receptor signaling pathway, NK cell-mediated cytotoxicity, etc. (**Figure 3D**). The PPI network of the 165 IRDEGs was built (**Figure 3E**). These results suggest that there is a significant imbalance of immune genes and pathways in the MCI patients.

### Screening of hub IRDEGs and their correlation with immune cell abundance

**Figure 4:**
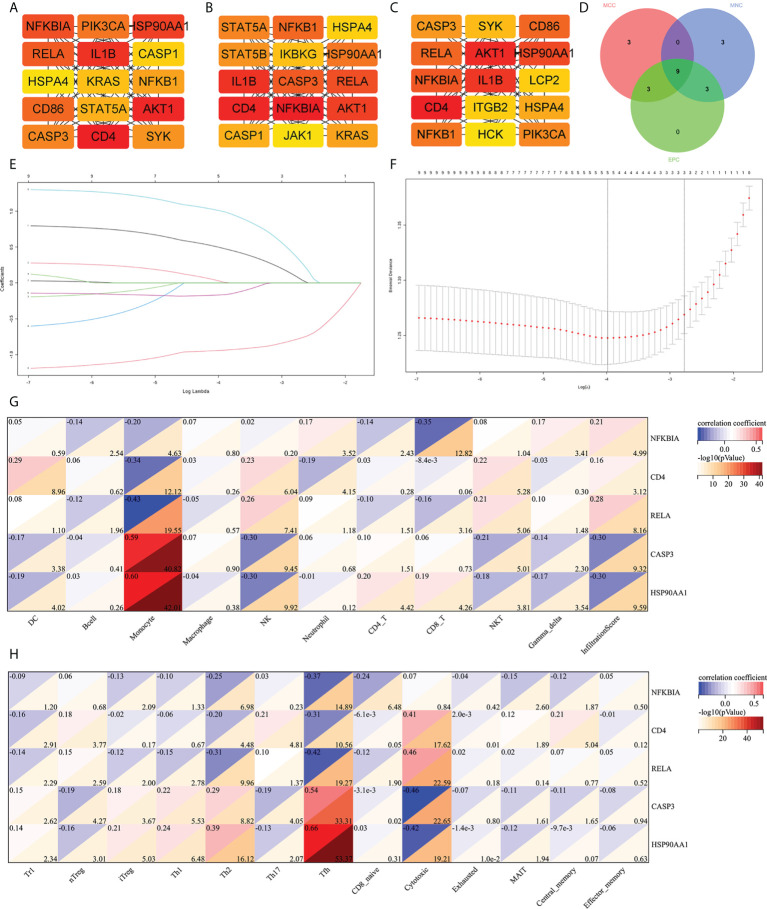
Screening of hub IRDEGs and their correlation with immune cell abundance. **(A–C)** The top 15 hub IRDEGs were screened by the EPC, MCC, and MNC algorithms of the cytoHubba plugin. **(D)** Venn diagram showing the common hub IRDEGs screened out by the EPC, MCC, and MNC algorithms. **(E)** LASSO coefficient profiles of the five genes that met the prognostic criteria initially. **(F)** The misclassification error in the jackknife rate analysis. **(G, H)** The heat map showed the correlation between five hub IRDEGs and 24 types of immune cell abundance and infiltration score. IRDEGs, immune-related different expression genes; HC, healthy control; MCI, mild cognitive impairment; AD, Alzheimer’s disease.

The cytoHubba was used to screen out the top 15 hub IRDEGs through MCC, EPC, and MNC algorithms (**Figures 4A–C**). The common part of the three algorithms was finally selected as the hub IRDEGs, including NFKBIA, CD4, IL1B, AKT1, RELA, CASP3, and NFKB1, HSP90AA1, and HSPA4 (**Figure 4D**). The expression profile of the above nine hub IRDEGs was then used to establish the LASSO model. Ultimately, five hub IRDEGs were retained, including NFKBIA, CD4, RELA, CASP3, and HSP90AA1 (**Figures 4E, F**). After that, we further analyzed the correlation between five hub IRDEGs and peripheral immune cell abundance to explore their regulatory effect on immune cell abundance. The result showed that the abundance of monocytes had the highest correlation with the expression level of five hub IRDEGs, which was positively correlated with the expression level of CASP3 and HSP90AA1, but negatively correlated with the expression level of NFKBIA, CD4, and RELA (**Figure 4G**). In addition, the correlation between the infiltration score and the expression level of five hub IRDEGs was statistically significant (**Figure 4G**). In the CD4 and CD8 subtypes, the abundance of expression of five hub IRDEGs was significantly correlated with the abundance of Th2, Tfh, and cytotoxic T cells (**Figure 4H**). Among them, Tfh abundance had the most significant correlation with five hub IRDEG expression levels.

### Verification of the expression level of hub IRDEGs

**Figure 5:**
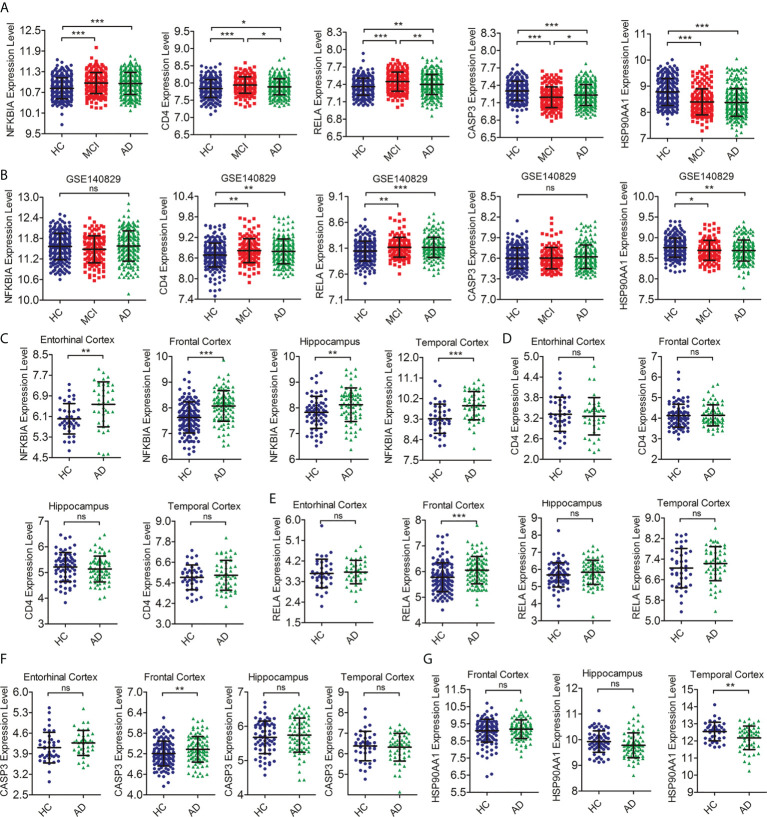
Verification of the expression level of hub IRDEGs. **(A)** The scatter plot showed the difference in expression levels of five hub IRDEGs in the peripheral blood of HC, MCI, and AD patients from the GSE63060 and GSE63061 datasets. **(B)** The scatter plot showed the difference in expression levels of five hub IRDEGs in the peripheral blood of HC, MCI, and AD patients from the GSE140829 datasets. **(C–G)** The expression level of five hub IRDEGs in four brain regions (entorhinal cortex, hippocampus, temporal cortex, and frontal cortex) of HC and AD groups were analyzed from the Alzdata database. The data were presented as mean ± SEM. The difference among the three groups was analyzed through a one-way analysis of variance (ANOVA) and followed by Fisher’s least significant difference (LSD) test. The difference analysis between the two groups was completed by a two-tailed Student’s *t*-test. ^*^ *p* < 0.05; ^**^ *p* < 0.01; ^***^ *p* < 0.001; ns, no statistical difference; IRDEGs, immune-related different expression genes; HC, healthy control; MCI, mild cognitive impairment; AD, Alzheimer’s disease.

To further detect the reproducibility of the selected hub IRDEGs, we further analyzed their expression levels in AD patients from the training dataset and the differences in expression levels between the HC, MCI, and AD groups in independent clinical cohorts (GSE140829). The results showed that the expression levels of five hub IRDEGs differed significantly between the AD and HC groups, which showed a similar trend to the MCI group (**Figure 5A**). In the independent cohort, the expression levels of CD4, RELA, and HSP90AA1 in the HC, MCI, and AD groups were significantly different, which was consistent with the training data (**Figure 5B**). In addition, we further examined their expression in the brain to determine the consistency of peripheral and CNS expression. The expression level of NFKBIA in the AD group was significantly higher than in the HC group in the entorhinal cortex, hippocampus, temporal cortex, and frontal cortex (**Figure 5C**). There was no significant difference in CD4 expression between the HC and AD groups (**Figure 5D**). The expression levels of RELA and CASP3 were increased in the AD group in the frontal cortex (**Figures 5E, F** **)**. The expression levels of HSP90AA1 was significantly decreased in the AD group compared with the HC group in the temporal cortex (**Figure 5G**). In summary, RELA and HSP90AA1 were finally identified as the two most reliable hub IRDEGs by verifying the RNA expression profile data in peripheral blood and brain tissues.

### GSEA analysis of hub IRDEGs and their correlation with AD-related pathological features

**Figure 6:**
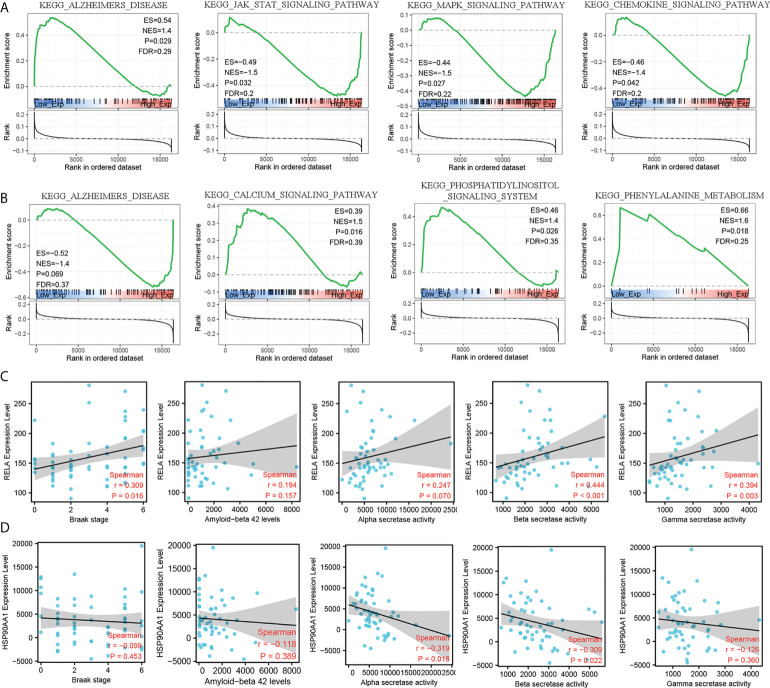
GSEA analysis of hub IRDEGs and their correlation with AD-related pathological features. **(A)** GSEA analysis of RELA. **(B)** GSEA analysis of HSP90AA1. **(C)** Spearman’s correlation analysis between the RELA expression level and Braak stage, α-, β-, and γ-secretase activity, and Aβ_42_ levels. **(D)** Spearman’s correlation analysis between the HSP90AA1 expression level and Braak stage, α-, β-, and γ-secretase activity, and Aβ_42_ levels. ES, enrichment score; NES, normalized enrichment score; FDR, false discovery rate; GSEA, gene set enrichment analysis.

To further explore the molecular mechanism of RELA and HSO90AA1, we selected the expression profile data of MCI patients from the training dataset for GSEA analysis. It was noteworthy that the expression levels of RELA and HSP90AA1 were enriched in Alzheimer’s disease pathways, suggesting their potential important roles in the AD process (**Figures 6A, B**). Furthermore, the Jak-Stat signaling pathway, MAPK signaling pathway, and chemokine signaling pathway were significantly enriched in the group with RELA high expression levels (**Figure 6A**). The HSP90AA1 expression level was related to the calcium signaling pathway, phosphatidylinositol signaling system, and phenylalanine metabolism (**Figure 6B**). The AD-related pathological features and expression profile data from GSE106241 were used to detect the correlation between hub IRDEGs and AD pathological features. The expression level of RELA was positively correlated with the BRAAK stage, α-, β-, and γ-secretase activities, but not with the level of Aβ_1-42_ (**Figure 6C**). HSP90AA1 expression level was negatively correlated with α- and β-secretase activity but not with BRAAK stage, Aβ_1-42_ level, or γ-secretase activity (**Figure 6D**).

## Discussion

Alzheimer’s disease is a progressive, age-related neurodegenerative disease. At present, its pathogenesis is not clear, and there is no disease-modifying therapy. In recent years, considerable evidence indicated that immune system dysfunction was a cardinal feature of AD pathogenesis and progression (19). More importantly, not only the immune process of the CNS but also the peripheral immune system dynamically alters and interacts with the central immune response in AD (20). Therefore, the rounded analysis of the abundance of peripheral immune cells and the consistency with the central immune system in AD, especially in the MCI stage, will provide a deeper understanding of the role of the immune system in the pathology and progression of AD and provide a new target for the early diagnosis and treatment of AD.

In this study, we analyzed the relative abundance of immune cells in the peripheral blood of HC, MCI, and AD patients. Infiltration scores representing the total abundance of immune cells were significantly higher in the MCI and AD groups than in the HC, while there was no difference between MCI and AD. This indicated that peripheral immune system alteration had occurred during the MCI stage. Similarly, MCI patients have also detected an upregulation level of neuroinflammation (10). Thus, alterations in the immune system were an early event in AD progression. In the peripheral innate immune cells, compared with the HC group, the abundance of DC, NK, and γδT cells was increased in the MCI group, while the abundance of monocyte was decreased. The AD group showed a similar trend to the MCI group. A recent study has found that the monocyte count in peripheral blood of AD patients was reduced and negatively correlated with the levels of AD-related pathological markers in the cerebrospinal fluid, including t-tau, p-tau, and tau/Aβ42 (21). Moreover, peripheral monocytes have been shown to participate in the phagocytosis of Aβ, and their phagocytosis of Aβ was reduced in AD (22). The function of NK cells, a subtype of innate lymphoid cells, in AD is not well understood. A previous study has found the activation of NK cells in peripheral blood of patients with MCI but not in mild AD (23). NK cell depletion can improve the cognitive function of 3xTg-AD mice by inhibiting neuroinflammation and promoting neurogenesis without influencing Aβ pathology (24). Dendritic cells (DC) are the bridge between innate immunity and adaptive immunity. In AD patients, a reduction in the abundance and functionality of DCs was observed (2). The strategy of combined intervention with bone marrow-derived DC stimulated by Aβ_1-42_ and splenocytes from young mice had a better effect on reducing Aβ load and improving cognitive levels in AD mice (25). In this study, we observed an increased abundance of DC in MCI patients, which may be related to the compensatory protective effect of DC in the early stages of the disease. As for γδT cells, Briggs et al. reported that γδT cells accumulated in the brain of 3xTg-AD mice and secreted IL-17 to trigger synaptic dysfunction (26). Therefore, the high abundance of γδT cells found in the MCI and AD groups in this study may accelerate the AD process.

The adaptive immune cells include B cells and T cells. There is strong evidence that adaptive immune cells also play an important role in AD (27). In this study, the abundance of B cells decreased in AD groups compared with HC and MCI groups. Previous studies have reported a decreased number of B cells in AD patients but an increase in IgG production (28). This was consistent with our results. As for T cells, we found that CD4 T cells decreased in MCI groups, while CD8 T cells showed no statistically significant difference. In CD4 T-cell subtypes, the abundance of iTreg, Th2, and Tfh decreased in the MCI and AD groups, while a decreased abundance of naïve CD4 T cells and Th1 was only detected in the MCI group. Under certain stimuli, naïve CD4 T cells can differentiate into antigen-specific T effectors, such as Th1, Th2, Th17, and Tregs (29). This may explain the decrease of CD4 T-cell subtypes in the MCI and AD groups in this study. In addition, the balance between Th1 and Th2 was an important regulator of immune homeostasis, and the imbalance of the Th1-to-Th2 ratio was a crucial event in neurodegenerative diseases (30). In this study, although the proportion of Th1 cells in the MCI group was reduced, the reduction of Th2 was even lower. This may lead to a decrease in anti-inflammatory cytokines and ultimately accelerate the progress of AD. In the early stage of AD, upregulation of Treg levels can restore the cognitive impairment of APP/PS1 mice by promoting microglial phagocytosis of Aβ (31). This suggests that this Treg has a beneficial effect in AD. In CD8 T-cell subsets, we found that cytotoxic T cells increased in MCI groups while MAIT was only decreased in the AD groups. In 2020, Gate et al. confirmed that the abundance of CD8^+^ T effector memory CD45RA^+^ cells was increased in the CSF and periphery of patients with MCI and AD (32). Furthermore, the proportion of cytotoxic T cells in the group with a high Aβ load was upregulated, suggesting a potential role in Aβ neuropathological mechanism (33). We found that there were many abnormalities in immune-related pathways in the MCI group, such as TNF-α, NK-KB, MAPK, and TGF-β signaling pathways. Dysregulation of these pathways was associated with T-cell and B-cell receptor signaling pathways, NK cell-mediated cytotoxicity, Th1, Th2, and Th17 cell differentiation. There were consistent with our analysis of differences in immune cell abundance between HC, MCI, and AD groups.

At the molecular level, we identified two immune hub genes (RELA and HSP90AA1) with similar differential expression in both peripheral blood and brain between HC, MCI, and AD groups. The v-rel avian reticuloendotheliosis viral oncogene homolog A (RELA) gene was a member of the NF-kB family, encoding the major transactivating subunit of the NF-kB protein complex (34). After a variety of stimuli, the RELA induced activation to take part in serious biological processes, such as immunity, cellular proliferation, differentiation and growth, apoptosis, etc. (35). In AD, NF-kB may be activated by Aβ to participate in the pathological process of AD, such as glial activation, neuronal apoptosis, and blood–brain barrier disruption (36). Similarly, we also confirmed that the RELA expression level was significantly increased in the blood and brain of AD patients, and this phenomenon already existed in the MCI stage. The expression level of RELA was positively correlated with Tau and Aβ pathology. Therefore, the activation of NF-κB pathway induced by RELA was very important in the early stage of AD. The results of the GSEA analysis confirm our results and those of previous studies. RELA expression level was closely associated with Alzheimer’s disease, JAK-STAT, MAPK, and chemokine signaling pathways.

Heat shock protein 90 kDa alpha family class A member 1 (HSP90AA1) gene locates the chromosomes 14q32.2 and encodes Heat shock protein 90α (HSP90α) (37). HSP90AA1 was essential for the homeostasis of its client proteins by taking part in their manipulation, folding, assembly, and degradation (38). More importantly, many client proteins of HSP90AA1 were involved in tumor pathogenesis, so HSP90AA1 played an important role in regulating the cancer cells’ growth and survival (38). In idiopathic Parkinson’s disease, HSP90AA1 was identified as a microglia activation marker and involved in the cellular response to cytokine stimulus, response to unfolded protein, and cytokine-mediated signaling pathway (39). In this study, we found that the expression level of HSP90AA1 was decreased in both MCI and AD, and was negatively correlated with α and β secretase activities. In addition, GSEA results confirmed that HSP90AA1 expression level was closely correlated with Alzheimer’s disease, calcium signaling pathway, phenylalanine metabolism, etc. These results provide new insights into the role of HSP90AA1 in AD.

This study had several limitations. Firstly, the abundance of immune cells in the peripheral blood of MCI and AD patients in this study was evaluated by a bioinformatics algorithm and was not verified by flow cytometry or single-cell sequencing technology. Second, this study focuses on the transcriptome level, which will be completed at the protein level in the future. Third, we will further explore the molecular mechanism of hub IRDEGs for AD *in vivo* and *in vitro*.

## Conclusions

In this study, we found that the alteration in peripheral immune cell abundance was an early event of AD that had occurred during the MCI stage. In addition, we identified RELA and HSP90AA1 as the immune hub genes related to MCI, with consistent expression trends in peripheral blood and the brain. It provided an important target for the early diagnosis and treatment of AD.

## Data availability statement

The original contributions presented in the study are included in the article/**Supplementary Material**. Further inquiries can be directed to the corresponding authors.

## Author contributions

H-DT and S-DC designed the study and prepared the manuscript. X-HQ and X-LL developed the methodology and analyzed the data. All authors contributed to the article and approved the submitted version.

## Funding

This work was supported by the National Natural Science Foundation of China [81971014] and the Shanghai Municipal Commission of Health and Family Planning [20184Y0056].

## Acknowledgments

We sincerely appreciate the high-quality data provided by the GEO and Alzdata databases.

## Conflict of interest

The authors declare that the research was conducted in the absence of any commercial or financial relationships that could be construed as a potential conflict of interest.

## Publisher’s note

All claims expressed in this article are solely those of the authors and do not necessarily represent those of their affiliated organizations, or those of the publisher, the editors and the reviewers. Any product that may be evaluated in this article, or claim that may be made by its manufacturer, is not guaranteed or endorsed by the publisher.

## Supplementary material

Supplementary Figure 1 Flow Chart of this study. HC: healthy control, MCI: mild cognitive impairment, AD: Alzheimer’s disease, WGNCA: Weighted Correlation Network Analysis, DEGs: differentially expressed genes, GO: Gene Ontology, KEGG: Kyoto Encyclopedia of Genes and Genomes, LASSO: least absolute shrinkage and selection operator, EC: entorhinal cortex, HP: hippocampus, TC: temporal cortex, FC: frontal cortex, GSEA: Gene Set Enrichment Analysis.
