# Supplementary figures and images for: Integrating peripheral blood and brain transcriptomics to identify immunological features associated with Alzheimer’s disease in mild cognitive impairment patients
Source: Front Immunol. 2022 Sep 9;13:986346. doi: 10.3389/fimmu.2022.986346 (PMC9501700; doi:10.3389/fimmu.2022.986346)

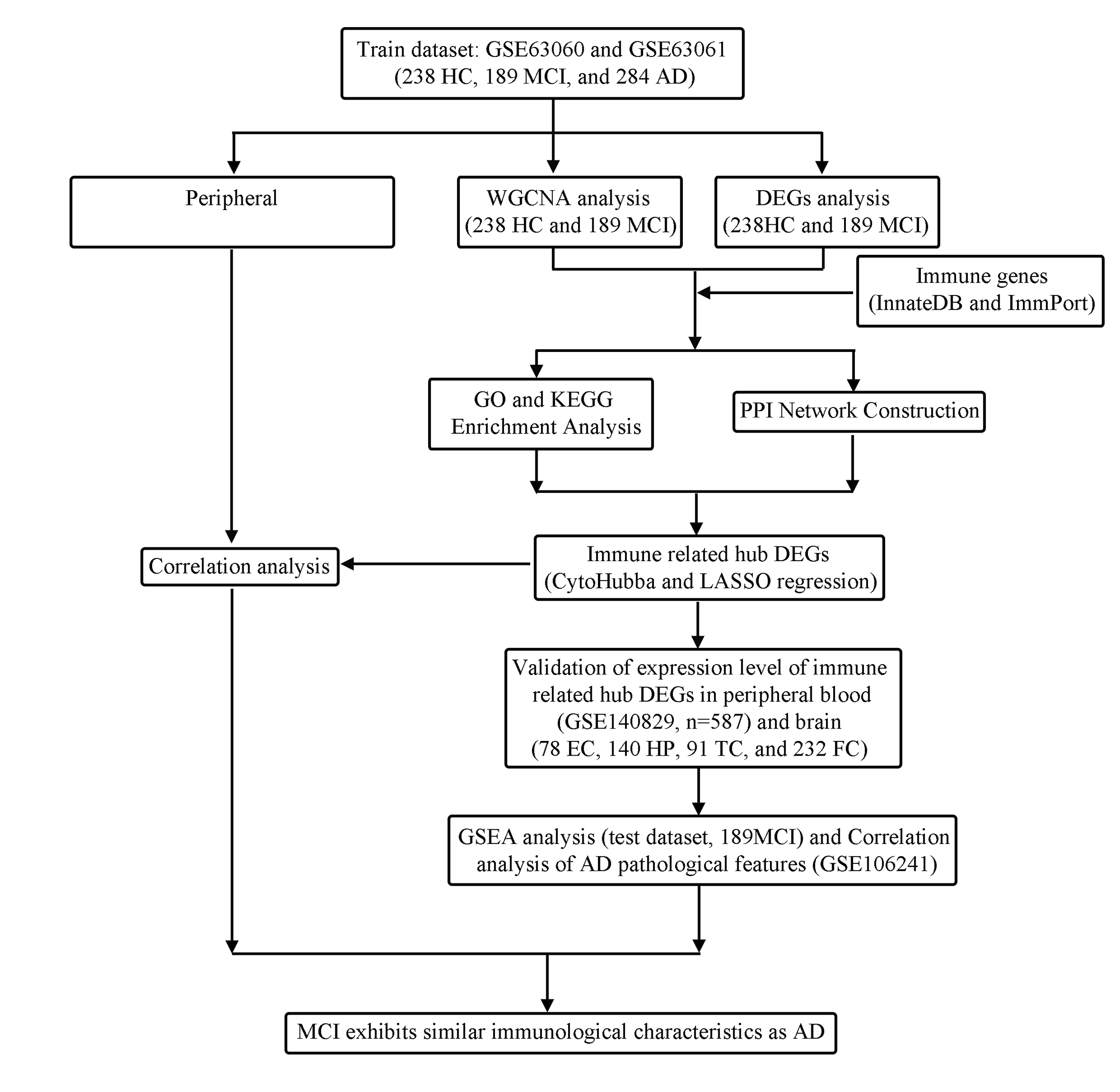

Supplement: Supplementary Figure 1 — Flow Chart of this study. HC: healthy control, MCI: mild cognitive impairment, AD: Alzheimer’s disease, WGNCA: Weighted Correlation Network Analysis, DEGs: differentially expressed genes, GO: Gene Ontology, KEGG: Kyoto Encyclopedia of Genes and Genomes, LASSO: least absolute shrinkage and selection operator, EC: entorhinal cortex, HP: hippocampus, TC: temporal cortex, FC: frontal cortex, GSEA: Gene Set Enrichment Analysis. [file Image_1.tif]
